# Brain activation induced by chronic psychosocial stress in mice

**DOI:** 10.1038/s41598-017-15422-5

**Published:** 2017-11-08

**Authors:** Mikaela A Laine, Ewa Sokolowska, Mateusz Dudek, Saija-Anita Callan, Petri Hyytiä, Iiris Hovatta

**Affiliations:** 1 Department of Biosciences, University of Helsinki, Helsinki, Finland; 2 Department of Pharmacology, University of Helsinki, Helsinki, Finland

## Abstract

Chronic psychosocial stress is a well-established risk factor for neuropsychiatric diseases. Abnormalities in brain activity have been demonstrated in patients with stress-related disorders. Global brain activation patterns during chronic stress exposure are less well understood but may have strong modifying effects on specific brain circuits and thereby influence development of stress-related pathologies. We determined neural activation induced by chronic social defeat stress, a mouse model of psychosocial stress. To assess chronic activation with an unbiased brain-wide focus we used manganese-enhanced magnetic resonance imaging (MEMRI) and immunohistochemical staining of ∆FOSB, a transcription factor induced by repeated neural activity. One week after 10-day social defeat we observed significantly more activation in several brain regions known to regulate depressive and anxiety-like behaviour, including the prefrontal cortex, bed nucleus of stria terminalis, ventral hippocampus and periaqueductal grey in stressed compared to control mice. We further established that the correlation of ∆FOSB positive cells between specific brain regions was altered following chronic social defeat. Chronic activation of these neural circuits may relate to persistent brain activity changes occurring during chronic psychosocial stress exposure, with potential relevance for the development of anxiety and depression in humans.

## Introduction

Psychosocial stress increases the risk for both somatic and psychiatric diseases and worsens their prognosis^1–4^. This term refers to a broad range of experiences, including lack of normative care, abuse, and problems in peer relationships such as bullying^5^. Stress-related mental disorders like major depressive disorder (MDD) and anxiety disorders are associated with altered activation of specific brain regions. For example, neuroimaging has revealed that resting-state activity is reduced in the prefrontal cortex (PFC)^6^ and increased in the hippocampus^7^ while task-related reactivity is decreased in the hippocampus^8^ and nucleus accumbens^9^ of MDD patients. Interpretation of the human imaging findings is difficult as activation patterns in tasks carried out during brain imaging may differ from those occurring during naturalistic chronic stress exposure. Because patients enrolled in these studies also tend to have an extensive history of symptoms and medication use, it is challenging to reveal direct consequences of chronic psychosocial stress itself. In contrast, animal models allow investigation of specific stressors in a controlled manner to establish the mechanisms connecting chronic psychosocial stress exposure, brain activation, and subsequent emotional and social behaviour.

Chronic social defeat stress (CSDS) is a rodent model of psychosocial stress, which produces many behavioural features similar to the symptoms of human anxiety and mood disorders, such as social aversion, anhedonia (decreased sucrose-preference) and increased self-administration of drugs of abuse^10,11^. These behavioural phenotypes last for several weeks post-stress^10^, and can be reversed by chronic treatment with selective serotonin re-uptake inhibitors or tricyclic antidepressants^12^, or a single dose of ketamine^13^. Thus, CSDS is an excellent mouse model to determine psychosocial stress-induced chronically occurring neural activation patterns, which may underlie the behavioural outcomes of stress exposure.

We hypothesized that CSDS induces neural activation in an extensive network of brain regions, including those previously implicated in acute stress and stress-related psychiatric disorders. We employed a combination of two methods for assessing neural activation in mice: manganese-enhanced magnetic resonance imaging (MEMRI) and ∆FOSB immunohistochemistry. MEMRI is based on the influx of paramagnetic Mn^2+^ ions into excitable cells through voltage-gated Ca^2+^ channels during depolarization, which increases T1-weighted signal intensity and thereby provides a marker of activated neurons^14,15^. Due to the slow efflux of Mn^2+^ from the brain, MEMRI allows functional brain imaging in anesthetized animals that have previously undergone pharmacological or behavioural testing^16,17^. In addition, Mn^2+^ ions are transported along axons, released from the terminals into the synaptic cleft, and taken up by the postsynaptic neuron^18,19^, providing a global measure of network activation.

ΔFOSB is a truncated hyperstable splice variant of the transcription factor FOSB. While the parent isoform FOSB is rapidly induced and degraded, the ∆FOSB form accumulates through repeated stimuli during a course of a chronic treatment, with each acute stimulus inducing a low level of ΔFOSB, and the repeated stress exposure causing the gradual increase in its total levels^20^. Since the estimated half-life of ΔFOSB is 8 days it is a useful marker for repeatedly activated neurons^20–22^.

In contrast to MEMRI that enables brain-wide mapping of neural activation and activity-dependent connectivity, ∆FOSB immunohistochemistry detects nuclear activation with high cellular resolution. By combining these methods, we compared CSDS-exposed and control mice one week after cessation of stress to determine brain regions activated due to chronic psychosocial stress exposure.

## Results

### Functional MEMRI mapping shows widespread brain activation by CSDS

**Figure 1:**
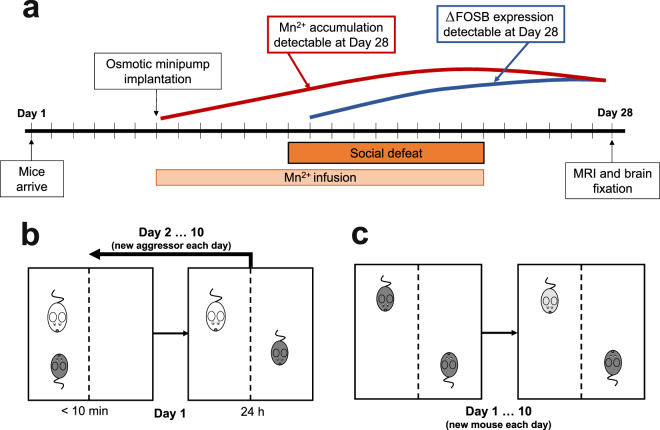
Timeline of the experiment and illustration of the chronic social defeat stress (CSDS) protocol. (**a**) Timeline of CSDS, manganese-enhanced magnetic resonance imaging and tissue collection. Each perpendicular line indicates one day. Estimated timeline for detection of Mn^2+^ by MEMRI and ∆FOSB by immunohistochemical staining is indicated by red and blue lines, respectively. (**b**) The experimental mouse in the defeated group (dark grey) was placed into the cage of a resident aggressor mouse (white) for a maximum of 10 minutes. It was then placed into the adjacent compartment of the same cage for 24 hours, retaining sensory contact without physical contact. The procedure was repeated for 10 days, each day in a novel resident aggressor mouse’s cage. (**c**) The control mouse was placed in a compartment adjacent to another control mouse, allowing sensory but not physical contact, for 24 hours. Each day the control mouse was placed into another cage with a novel control mouse, and the procedure was repeated for 10 days. MRI = magnetic resonance imaging.

**Figure 2:**
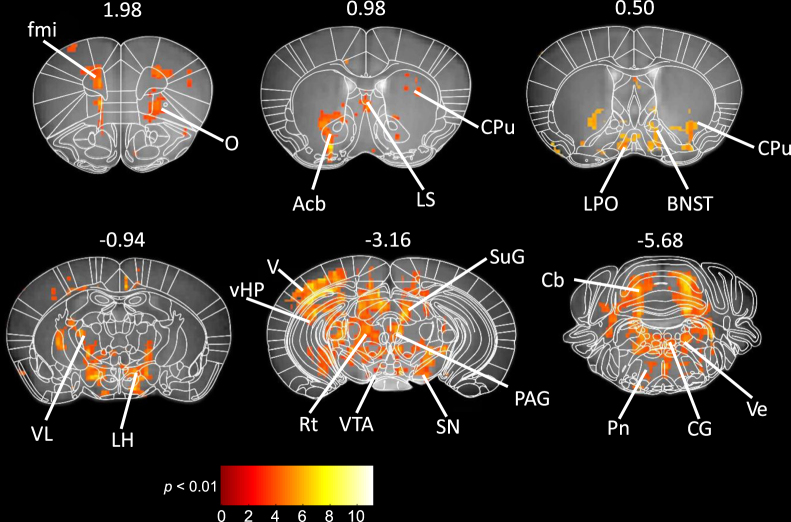
Differences in brain activity between socially defeated and control mice revealed by MEMRI. Statistical t-maps (thresholded at *p* < 0.01, corrected for multiple comparisons) are superimposed on a custom-made mouse brain template, with corresponding atlas sections^58^ manually overlaid using anatomical landmarks. Numbers indicate the positions of the sections from bregma in millimetres. Brain region abbreviations: BNST (bed nucleus of the stria terminalis), CPu (caudate putamen), Cb (cerebellum), CG (central grey), fmi (forceps minor), LH (lateral hypothalamus), LPO (lateral preoptic area), LS (lateral septum), Acb (nucleus accumbens), O (orbital cortex), PAG (periaqueductal grey), Pn (pontine nuclei), Rt (reticular formation), SN (substantia nigra), SuG (superior colliculus), V (visual cortex), VL (ventrolateral thalamic nucleus), vHP (ventral hippocampus), Ve (vestibular nuclei), VTA (ventral tegmental area).

We exposed mice to the 10-day CSDS protocol, during which MnCl_2_ was infused from an implanted osmotic minipump (Fig. 1). Socially defeated mice showed significant enhancement of T1 signal intensity in MEMRI compared to control mice as shown by statistical parametric maps in Fig. 2. Enhanced T1 intensity reflects accumulation of Mn^2+^ into repeatedly activated brain regions, suggesting increased brain activity in mice exposed to CSDS. We did not detect signal decreases in stressed mice compared to the controls. It is possible that this is due to the principle of MEMRI, i.e., Mn^2+^ influx into active neurons by voltage-gated Ca^2+^ channels, which renders the method less sensitive to deactivation than activity-induced signal increase. We observed increased activation throughout the brain, mostly confined to specific anatomical regions. In the PFC, increased signal was found in the orbital cortex (O) and forceps minor (fmi), a white matter tract. More caudally, the nucleus accumbens (Acb), caudate putamen (CPu), and lateral septum (LS) exhibited increased activation. Both the striatal and septal clusters were also activated by chronic stress in more caudal sections. In the basal forebrain, ventral parts of the bed nucleus of the stria terminalis (BNST) and preoptic area (LPO) were more activated in stressed compared to the control mice. The preoptic activation continued caudally as a distinct activation cluster encompassing the lateral hypothalamus (LH) with ventrolateral thalamic (VL) connections. We detected stronger MEMRI signal in the midbrain, including the ventral tegmental area (VTA), substantia nigra (SN), periaqueductal grey (PAG), superior colliculus (SuG), ventral hippocampus (vHP), dentate gyrus (DG), and reticular formation (Rt) in stressed compared to the control mice. Cortical activation was found in the visual cortex (V). In the hindbrain, social defeat was associated with increased signal intensity in the pontine (Pn) and vestibular nuclei (Ve), with connectivity with the cerebellum (Cb).

### ∆FOSB staining reveals localised cell populations with repeated activation in stressed mice

**Figure 3:**
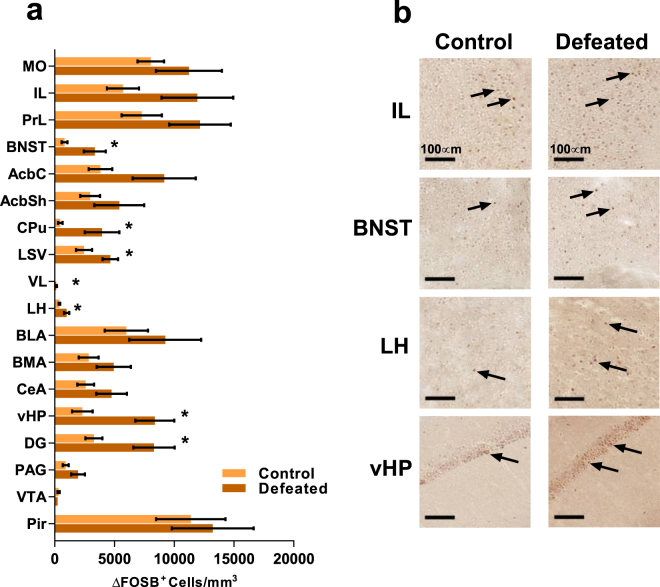
Chronic social defeat induces changes in brain activation as measured by expression of ∆FOSB. (**a**) The mean number of cells staining positive for ∆FOSB in defeated and control mice. Error bars ± 1 SEM, *p < 0.05, see Table 1 for exact statistical values. (**b**) Representative images of immunohistochemical staining for ∆FOSB, positive cells indicated by black arrows, in the infralimbic cortex (IL), bed nucleus of stria terminals (BNST), lateral hypothalamus (LH) and ventral hippocampus (vHP). Scale bar = 100 µm. See Supplementary Fig. S2 for representative images from each of the brain regions analysed.

We counted the number of ∆FOSB-expressing cells in 18 pre-selected brain regions and compared the control and defeated groups with either *t*- or *U*-tests depending on normality of distributions (Table 1). We selected the regions based on our results from MEMRI and on prior findings on brain activation in stress and emotional processing^23,24^. As shown in Fig. 3, we observed significantly larger numbers of ∆FOSB positive cells in the BNST (anterodorsal), CPu, lateral septum (ventral, LSV), lateral thalamus (VL), LH, vHP and the DG (ventral) in stressed compared to control mice. While no comparisons survived correction for a false discovery rate (FDR) of 5%, given the large number of regions examined, the aforementioned regions remained significant when correcting for FDR < 20%. The number of ∆FOSB positive cells in midbrain regions such as the VTA and PAG (dorsolateral) was overall very low, and we did not detect differences in their number between stressed and control animals in either region (Fig. 2A). Expression of ∆FOSB in the infralimbic (IL) and prelimbic (PrL) regions of the prefrontal cortex did not differ significantly between groups (Table 1). No differences between groups were observed for the negative control region piriform cortex (Pir), as expected, as it does not have a known role in the regulation of stress. There were very few ∆FOSB-positive cells in the CPu, VL and LH, especially in the VL, prohibiting calculation of the percentage change between groups.

**Table 1:** Differences in the number of ∆FOSB-positive cells between defeated and control mice in each brain region of interest.

Brain region	Full name	*N* control	*N* defeated	*% change* ^+^	*t/U*	*P*
MO	*Medial orbital cortex*	7	7	+39.7	1.072 (*t*)	0.315
IL	*Infralimbic cortex*	7	8	+109.1	1.801 (*t*)	0.095
PrL	*Prelimbic cortex*	7	8	+66.9	1.536 (*t*)	0.148
BNST	*Bed nucleus of stria terminalis (anterodorsal)*	6	7	+306.4	2.644 (*t*)	**0.034**
AcbC	*Nucleus accumbens (core)*	7	8	+139.4	1.389 (*U*)	0.189
AcbSh	*Nucleus accumbens (shell)*	6	8	+82.3	0.645 (*U*)	0.573
CPu	*Caudate putamen*	7	8	+753.7	2.083 (*U*)	**0.006**
LSV	*Lateral septum (ventral)*	7	8	+89.0	2.142 (*t*)	**0.035**
VL	*Thalamus (ventrolateral)*	6	7	n/a	2.448 (*U*)	**0.035**
LH	*Lateral hypothalamus*	6	7	+153.1	2.436 (*t*)	**0.033**
BLA	*Basolateral amygdala*	6	7	+54.2	0.886 (*t*)	0.394
BMA	*Basomedial amygdala*	6	7	+73.5	1.206 (*t*)	0.253
CeA	*Central amygdala*	6	7	+83.7	1.410 (*t*)	0.186
vHP	*Ventral hippocampus (CA1 & CA3)*	7	8	+261.5	2.083 (*U*)	**0.040**
DG	*Dentate gyrus (ventral)*	6	6	+153.4	2.695 (*t*)	**0.032**
PAG	*Periaqueductal grey (dorsolateral)*	7	8	+112.8	1.582 (*t*)	0.138
VTA	*Ventral tegmental area*	6	6	−22.7	0.601 (*t*)	0.561
Pir	*Piriform cortex*	7	8	+16.1	0.401 (*t*)	0.695

The number of positive cells between control and defeated mice was compared with either an independent Student’s *t*-test (normally distributed data, indicated with *t*) or a Mann-Whitney U-test (non-normally distributed data, indicated with *U*). *N* = number of animals, *P* = nominal *p*-value, +change in mean number of stained cells in defeated mice compared to controls in percentage points. Significant *p*-values (<0.05) which also survive correction for multiple comparisons (false discovery rate < 20%) are marked in bold.

### CSDS alters the correlation of ∆FOSB expression between brain regions

**Figure 4:**
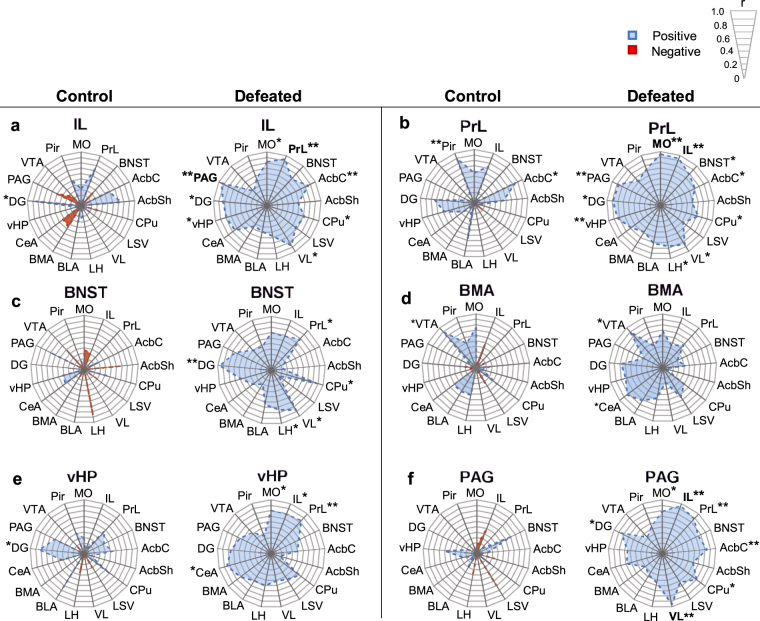
Chronic social defeat stress associates with alterations in ∆FOSB positive cell number correlation profiles of specific brain regions. Radar graphs depict the absolute value of *r* for the infralimbic cortex (IL, a), prelimbic cortex (PrL, b), bed nucleus of stria terminalis (BNST, c), basomedial amygdala (BMA, d), ventral hippocampus (vHP, e), and periaqueductal grey (PAG, f) comparison of the number of ∆FOSB-positive cells per mm^3^ with the other brain regions. Figure key indicates the sign and magnitude of *r*. Regions that correlate significantly with the title region are indicated by an asterisk (*p < 0.05, **p < 0.01), and correlations remaining significant after correction for false discovery rate (<5%) are marked with bold text. See Table 1 for brain region abbreviations.

To identify putative network-level consequences of CSDS, we took advantage of the nuclear specificity and excellent spatial resolution of immunohistochemistry and calculated correlation coefficients (Pearson’s *r*) of the number of ∆FOSB positive cells between each brain region, separately for defeated and control mice (see Supplementary Table S1 for full correlation matrix). We observed notable differences in the correlation profiles of defeated and control mice for specific brain regions (Fig. 4). The correlation profiles of prefrontal regions IL and PrL were similar within the control group and the stressed group, but the two groups differed considerably from each other. The number of ∆FOSB positive cells correlated significantly (*r* > 0.8, *p* < 0.05) in both groups with expression in the DG and AcbC, while in defeated mice we also observed correlation of both regions with the CPu and VL (Fig. 4a and b). Activation of the BNST was also significantly correlated with the activation of subcortical structures, PrL and DG following CSDS but not in control mice (Fig. 4c). The basomedial amygdala (BMA, Fig. 4d) shared correlated activation with the VTA both in control and defeated mice. For the defeated mice, the only notable change in BMA co-activation occurred with other amygdalar nuclei, namely the central nucleus (CeA), with no significant correlations with frontal and other subcortical regions. Lastly, both the vHP (Fig. 4e) and PAG (Fig. 4f) cell counts were significantly correlated with frontal regions in defeated mice but not in controls, despite the very low number of ∆FOSB positive cells observed in the PAG. To control for potential biases in correlation coefficients caused by an overall increase in ∆FOSB expressing cells in stressed mice, we tested for a correlation of mean ∆FOSB cell number and mean value of *r* for each brain region, separately for the two groups. In defeated mice there was no correlation between ∆FOSB and *r*, suggesting that the emerging correlation profiles cannot be explained by a rise in absolute ∆FOSB expression (see Supplementary Fig. S1).

## Discussion

To determine mouse brain regions repeatedly activated due to chronic stress exposure, we carried out neuroimaging by MEMRI and immunohistochemical detection of ∆FOSB one week after the cessation of 10-day CSDS in mice. Our approach allowed unbiased covering of the whole brain, enabling the discovery of previously unreported associations between repeated neuronal activity and chronic stress experience. Regions in which both methods revealed significantly more activation in stressed compared to control mice included subcortical structures (BNST, CPu, LSV, VL, LH), and the vHP and DG. MEMRI revealed stress-induced activation at the orbital cortex and fmi, an anterior portion of the corpus callosum, possibly reflecting the spread of Mn^2+^ along white matter tracts^25^. A stronger MEMRI signal was also observed in the midbrain regions of stressed compared to control mice, but without any differences in the number of ∆FOSB positive cells between the groups. To assess putative stress effects at the network level, we also surveyed correlations of ∆FOSB expression between brain regions, separately within the defeated and control groups. The correlation profiles of the IL, PrL, BNST, vHP and PAG were considerably altered following CSDS.

Although both ∆FOSB expression and MEMRI mapping are expected to reveal differences in brain activation induced by chronic psychosocial stress, the observed regional pattern of activation was not completely overlapping. These findings are not contradictory, but rather reflect methodological differences, and represent complementary measures of repeated brain activation. While ∆FOSB immunohistochemistry allows measurement of high resolution neuronal activation, MEMRI can also reveal long-range connectivity in activated pathways due to anterograde axonal Mn^2+^ transport in addition to local activity^18,19^. Therefore, strong MEMRI signal can also be found in regions with low ∆FOSB expression levels but dense axonal tracts passing through them, as seen in VL, LH and midbrain regions. For example, the LH receives strong inputs from the extended amygdala, striatum, septum, and hippocampal formation, which had higher numbers of ∆FOSB positive cells in stressed compared to control mice. These regions also send reciprocal projections to the hypothalamus. In addition, the LH is connected with the midbrain and brainstem^26^ which had widespread MEMRI signal enhancement in stressed mice. These regions play an important role in somatomotor, sensory, and autonomic control mechanisms that have been implicated in stress and anxiety^27^.

Neither MEMRI nor ∆FOSB mapping allows us to determine the exact time of neuronal activation giving rise to the observed differences between stressed and control animals. However, with the 8-day half-life of ∆FOSB^21^, the number of ∆FOSB positive cells we observe one week after 10-day CSDS likely reflects repeated activation that occurred during defeat and/or the time between CSDS and dissection. The half-life of Mn^2+^ in the brain is longer than that of ∆FOSB, and therefore brain activation measured by MEMRI one week after social defeat may partly reflect acute stress effects that occurred during the first days of social defeat. However, we have previously shown that activation induced by a week of alcohol exposure either largely subsided or exhibited an altered pattern following a week-long abstinence from alcohol, suggesting that the changes seen during abstinence could not be ascribed to acute alcohol exposure alone^17^. Similarly, the MEMRI signal measured one week after CSDS could reflect neuronal activation related to long-term physiological and behavioral changes observed in defeated animals^10^.

The regions we selected for immunohistochemical analysis have been implicated in the processing of social and emotional behaviour in mice and humans^6,23,24,28^. However, we only observed repeated activation of a subset of these regions during chronic psychosocial stress in mice. These differences to previous publications are most likely due to varying nature of stress and the interval between the stress exposure and measurement of brain activation. Similarly to our findings, the dorsal BNST, CPu, LSV and DG were previously found to be activated already 24 h following CSDS in mice^29^, and the PFC, LH and DG were activated less than 24 h after chronic variable stress in rats^30^. No differences were observed in ∆FOSB expression 20 days post-defeat between the control and defeated mice using Western blot detection of FOSB isoforms in bulk tissue samples including the whole PFC and whole hippocampus^31^. Also the Acb, basolateral amygdala (BLA) and dorsal raphe nuclei were found to be activated 24 h following stress^29^, but not in our study with a longer post-defeat delay. These regions could therefore be involved more significantly during early stress exposure, as ∆FOSB produced in the early days but not later days of stress exposure may have decayed by the time we measured its expression. The regions displaying activation in our study may mediate the effects of chronic psychosocial stress in the long term and are potential sites of alterations that may also contribute to the development of behavioural outcomes of stress.

When two brain regions jointly participate in a task, their activity becomes correlated as measured by functional neuroimaging in humans^32,33^, electrical recordings in monkeys^34^ and staining for activity-induced proteins in rodents^35,36^. Although immunohistochemical data analysis reveals such functional connections, directionality of the connections remains elusive. Some regions are even known to be connected by parallel tracts with functionally opposed consequences on activation^37^. Furthermore, regions that engage in correlated activation are not necessarily physically connected, but rather part of the same functional network. Despite these limitations, we aimed at addressing functional connectivity for establishing how chronic psychosocial stress is processed by specific brain networks.

The BNST is implicated in risk assessment and anxiety-like behaviour in mice^38^, but its activation in CSDS has not been extensively studied. BNST neurons mediate anxiety-like behaviour and regulate endocrine functions following acute stress exposure^39^. Some of its subregions have opposing roles in anxiety-like behaviour: the anterodorsal nuclei produce anxiogenic responses, while the ventral nuclei mediate anxiolysis^23,37^. The dorsal segment shows increased activation 24 h following 10-day CSDS^29^. We found that the anterodorsal BNST is repeatedly activated in stress-exposed mice, and that in the stressed mice the BNST ∆FOSB expression correlation with other brain regions, including the DG, LH, VL, CPu and PrL, was altered. The BNST has afferent and efferent connections with several regions, including the amygdala, LH, PAG and the PFC^40–42^, and neural activity of BNST could partly contribute to increased MEMRI signal in these areas through axonal transport. However, out of these established connections we only observed significant correlation of BNST activation with the LH and the PrL following CSDS. The observed correlation between the BNST and DG is interesting because no direct structural connections are reported for these regions in mice^43^, suggesting that their functional connectivity may be mediated by one or more other regions. Therefore, while structural and functional connections of the BNST are wide-reaching, only some of these connections may be involved in the response to chronic psychosocial stress. In particular, our data suggest that networks connecting the BNST, PFC, LH and DG are activated during chronic stress, and may mediate previously unprobed long-term adaptive changes after stress.

The vHP and PFC both regulate emotionality and depressive states in humans. In patients with MDD the vHP is less active during a memory task^8^, but more active at rest^7^ compared to healthy controls, while the PFC exhibits decreased activity at rest^6^. Both regions are activated in humans during an acutely stressful task^44,45^. We observed repeated activation of both regions and a significant correlation of ∆FOSB expression between them in mice exposed to CSDS. In mice these regions are connected by a monosynaptic tract^46^, in addition to several indirect connective loops^47^. Temporally synchronized firing between these regions occurs during anxiety-provoking tasks^48^, and inhibiting activity along this pathway optogenetically reduces anxiety-like behaviour^49^. Interestingly, in humans the tract connecting the hippocampal region to the PFC, the uncinate fasciculus, shows decreased white matter integrity by diffusion tensor imaging in adults with a history of childhood maltreatment^50^. We observed that the fmi, another major white matter tract of the PFC, was more active in defeated than control mice as measured by MEMRI, suggesting that this connection also becomes recruited during chronic stress. White matter alterations have also been found in this tract in mice exposed to social isolation^51^ and in MDD patients^52^. Thus, chronic psychosocial stress in mice, and acute social stress in humans, both increase activity in the circuit involving the PFC and vHP.

We did not detect altered ∆FOSB expression in the PAG after stress, but observed marked changes in its correlation profile. This region contains connections to and from the cortex, basal ganglia, VL and LH^53^, and it is an important mediator of defensive behavioural responses via inputs from the amygdala^54^. Similarly to the BNST, only a subset of the regions with direct synaptic connectivity with the PAG showed significantly correlated activation in the stressed mice, including the PFC, AcbC, and VL. Anatomical connectivity of these regions with PAG may also lead to the increased Mn^2+^ accumulation we observed. The emergence of correlation between low PAG ∆FOSB expression and other regions suggests that the observed correlations were not artefacts arising from an increased number of ∆FOSB expressing cells (see Supplementary Fig. S1). Our finding of low ∆FOSB expression in the PAG parallel with significant functional connections may point to a small specific neuronal ensemble participating in chronic stress processing. Intriguingly, fewer than 10% of PAG neurons receive input from the cortex, and these cells mediate social behaviour following chronic stress exposure^53^.

In conclusion, our data suggest that a functional network of specific frontal, subcortical and midbrain regions is repeatedly activated by CSDS, and that chronic stress alters inter-region co-activation. These brain regions, including the PFC, BNST and other subcortical structures, vHP and PAG, provide targets for future studies seeking to determine long-lasting effects of chronic psychosocial stress on the mouse brain.

## Methods

### Animals

Animal procedures were approved by the project authorization board of the Regional State Administration Agency for Southern Finland and carried out in accordance to directive 2010/63/EU of the European Parliament and of the Council, and the Finnish Act on the Protection of Animals Used for Science or Educational Purposes (497/2013).

We used 21 male mice (C57BL/6NCrl, Charles River, Sulzfeld, Germany), aged 5 weeks on arrival. During a 7-day acclimatization period they were group-housed in Makrolon type III cages. The housing facility was temperature- and humidity-controlled with a 12 h light/dark cycle (light phase: 6:00–18:00). Mice had *ad libitum* access to standard pellet food and water.

Aggressor mice used for the chronic social defeat stress (CSDS) procedure (see below) were male Clr:CD1 (Charles River) mice aged between 13 and 26 weeks. Following a 7-day acclimatization period in single-housed individually ventilated cages we screened them for appropriate levels of aggression by assessing their propensity for attacking an intruder mouse (no sooner than 10 seconds or later than 90 seconds after introduction to a C57BL/6NCrl test mouse). Twenty-four hours before the start of CSDS each selected aggressor mouse was placed alone into a cage to be used for CSDS.

### Experimental design

The experimental procedures are illustrated in Fig. 1. At the end of the habituation period, mice were surgically implanted with osmotic minipumps for delivery of MnCl_2_. The mice were allowed to recover from the surgery for one week, after which they underwent the 10-day CSDS procedure or the control housing procedure. Manganese was infused constitutively during CSDS. After the end of CSDS, all mice were placed in single-housed cages until the MRI scan and dissections. We wanted to mainly assess chronic brain activation without bias from acute activation occurring at the beginning of the social defeat and therefore set the post-defeat delay to six days.

### Osmotic minipump implantation

Subcutaneous osmotic minipumps (Alzet, model 2002) delivering 200 µl of MnCl_2_ (0.5 µl/h) were implanted as described previously^55^. MnCl_2_ was dissolved into Tris-buffered saline (pH 7.4) and administered with osmotic minipumps during the 16-day infusion, corresponding to a total MnCl_2_ dose of 160 mg/kg/week. The concentration of MnCl_2_ in the pumps was adjusted to the body weight of the animals. Before the surgery, the pumps were primed overnight in a 37 °C saline solution. Animals were anesthetized with 5% isoflurane and the pumps were implanted subcutaneously on the dorsum, slightly caudal to the scapulae. For post-surgical analgesia, animals received one subcutaneous injection of carprofen (5 mg/kg) immediately after implantation.

### Chronic social defeat stress (CSDS)

Following recovery from implantation mice allocated to the defeated group (*N* = 11) were exposed to CSDS^56^. In short, the mouse was placed into a cage containing an aggressor mouse. The mice were observed for a maximum of 10 min, after which they were separated by a perforated plexiglass wall, exposing the experimental mouse to sensory cues from the aggressor mouse without risk of physical contact. If during the initial observation phase the aggressor mouse exhibited excessive physical aggression (caused a significant wound, particularly in the vicinity of the minipump) the mice were separated before the 10-minute time had elapsed. The average contact time throughout the experiment was 6.43 min. The sensory contact phase lasted for 24 h, after which the experimental mouse was placed into the cage of a novel aggressor mouse and the 10-min physical contact + 24 h sensory contact cycle was repeated. This procedure was repeated for 10 consecutive days. Control mice (*N* = 10) were housed in similar cages but with another control mouse as a neighbour and no physical contact, switching cage-mates daily. The social aversion test was performed 24 h after the last session of social defeat for both defeated and control mice^56^. Briefly, mice were placed in an arena containing a perforated cylinder for 150 s. The mice were placed back in the home cage, and the cylinder replaced with one containing a novel CD-1 mouse. The mouse was then returned to the arena for 150 s. The social preference score (ratio of time spent near the cylinder when it contained another mouse compared to the trial with the empty cylinder) did not differ (*p = *0.256) between the groups due to small sample size and large variation between animals.

### MRI acquisition and data analysis

Magnetic resonance imaging was performed with a 4.7 T scanner (Bruker, PharmaScan 47/16 US, Ettlingen, Germany) using a 38 mm linear volume coil for transmit and receive. Mice were transferred from the vivarium to the scanner room, immediately anaesthetized with 5% isoflurane in oxygen (0.8–1 L/min) and positioned on a custom-made holder with a stabilizing tooth bar and a nose cone. Accurate positioning was confirmed with the help of scout images and all inaccuracies were corrected by adjusting the position and angles of the field. During scanning anaesthesia was maintained with 2–3% isoflurane and the body temperature was kept constant at 37 °C with a heating pad. The scanning order was counterbalanced according to the experimental groups. T1-weighted images were acquired using a three-dimensional rapid acquisition-relaxation enhanced (RARE) pulse sequence (repetition time = 300 ms, effective echo time = 10.7 ms, echo train length = 8, flip angle = 180°, number of averages = 8, field of view FOV = 12.8 × 12.8 × 12.8 mm, acquired matrix size = 200/200/200, reconstructed matrix size = 100/100/100, resulting in 0.128 × 0.128 × 0.128 mm^3^ voxel resolution). Values for the automatically adjusted receiver gain were collected to verify that the observed group differences were not caused by changes in receiver gain. Total scanning time, including acquisition of scout images, was approximately 60 min per animal.

All MRI images were converted to Analyze format, scaled by a factor of 10 and spatially preprocessed with SPM8 software running under MATLAB (version R2015). All T1-weighted images were spatially normalized to a custom-made mouse brain template. Bias correction was performed to eliminate field inhomogeneity of the magnetic field. For creating statistical parametric maps of differential brain activation between experimental groups, the groups were compared by voxel-wise independent two-tailed *t*-test using SPM8. Despite careful adjustment of MnCl_2_ concentration in individual animals, systemic infusion can lead to differences in brain Mn^2+^ accumulation, causing activation-independent differences in the mean global signal intensity between subjects. To eliminate global effects, the mean intensity was included as a nuisance factor in the statistical model, on a voxel-by-voxel basis^57^. For the defeated < control contrast, the resulting statistical parametric maps were thresholded voxelwise at the arbitrary significance of *p* < 0.001. Then the cluster size was thresholded with a threshold of k = 71 voxels, resulting in an overall significance level of *p* < 0.01 corrected for multiple comparisons across the whole brain. k was computed based on Monte Carlo simulations. Brain regions were identified by superimposing brain atlas sections^58^ on the MRI scans using prominent landmarks, including white matter tracts (e.g. corpus callosum, anterior and posterior commissures), the overall shape of the brain and ventricle location, and defined grey matter structures (e.g. caudate putamen). The observed increased MEMRI signal in stressed animals is likely not attributable to alterations in Ca^2+^ channel function as we did not observe any gene coding for Ca^2+^ channel components (GO:0005891) to be differentially expressed between stressed and control animals in RNA sequencing data from the medial PFC or vHP (data not shown).

### Tissue processing and immunohistochemistry

Following the MRI scan mice were injected with a lethal dose of pentobarbital (Mebunat Vet, Orion Pharma) without breaks in anaesthesia. Mice were transcardially perfused with 4% paraformaldehyde (PFA) in PBS. We postfixed the brains in 4% PFA for 2–24 h, cryoprotected them in 20% sucrose for 24 h, froze them in 2-methyl butane, and stored them in −80 °C. We then cryosectioned the brains with a Leica CM3050 cryostat (Leica Biosystems, Nussloch, Germany). Sections were stored in −20 °C submerged in cryoprotectant. One defeated animal died during MRI acquisition and was not further analysed.

All immunohistochemical, microscopy and quantification procedures were carried out blind to the condition of each animal. Five animals (3 controls, 2 defeated) were not stained due to issues with fixation. Tissue sections were stained in batches with balanced numbers of mice belonging to each experimental group. The sections were rinsed in TBS and endogenous peroxidase activity was quenched with hydrogen peroxide. We blocked non-specific binding with 10% normal goat serum (NGS) with 0.5% Tween-20 detergent (Sigma Aldrich) in TBS. Primary antibody incubation with a polyclonal rabbit anti-FOSB antibody [1:500 (0.4 µg/mL), sc-7203, Santa Cruz Biotechnology, Dallas, TX, U.S.A.] in TBS with 10% NGS + 0.5% Tween-20 was carried out for 18 h in ambient room temperature. Biotinylated affinity-purified goat anti-rabbit [1:200 (7.5 µg/mL), ABC detection kit (PK-6101), Vector laboratories, Burlingame, CA, U.S.A] diluted in TBS with 1.5% NGS was used as the secondary antibody and incubated for 2 h in room temperature. The sections were incubated in an avidin-biotin solution prepared in PBS (ABC detection kit, Vector Laboratories), rinsed in 0.1 M PB and staining detected with 3,3′-diaminobenzidine (DAB, Vector Laboratories). Sections were dehydrated and stored at +4 °C.

The primary antibody detects also the full-length FOSB protein, but it is induced only by acute stimulation with a half-life of 1.6 hours^20,21^ and is thus not expected to contribute to our findings. As the mice were kept anaesthetized throughout the scanning, which lasted approximately 60 min, and the perfusion was done immediately after the scan, acute stress effects incurred during transport from the vivarium to the scanning room are expected to be minimal. Due to the counterbalanced order of scanning, any minor acutely induced full-length FOSB did not impact our results. The primary antibody was tested on male C57BL/6NCrl mice receiving injections of saline or cocaine for 6 consecutive days (15 mg/kg daily), and naïve controls (*N* = 2/group). Cocaine is known to induce both full-length and ∆FOSB in the caudoputamen after acute exposure, but only ∆FOSB following chronic exposure^59^. Mice receiving cocaine had significantly more ∆FOSB positive cells than both saline and naïve control mice (*p* < 0.001 in both contrasts, see Supplementary Fig. S1). Validity of staining was also ensured with test staining omitting the primary antibody.

Stained sections were imaged with a Pannoramic FLASH II digital scanner using a 0.8 NA objective at a resolution of 0.24 µm/pixel (3DHistech, Budapest, Hungary) at the Institute of Biotechnology (University of Helsinki). We captured regions of interest images from the digital scans at 20x magnification using the Pannoramic Viewer software version 1.15.3 (3DHistech) based on anatomical landmarks indexed in the Mouse Brain Atlas^58^. The numbers of stained cells were quantified using ImageJ version 1.47 v (National Institutes of Health), using a colour deconvolution plugin with a vector for DAB-staining built in to this version of the software. Thresholded images were analysed for particles larger than 15 µm^2^ in size and quantified with automated measurement.

### Cell count statistical analysis

∆FOSB cell counts were acquired from 18 relevant brain regions (see Table 1) from 3–6 sections from each animal. The piriform cortex (Pir), an olfactory processing region^60^, was included due to its expected lack of involvement in CSDS. Exclusion criteria for single cell count data points was a deviation of ≥3 times the interquartile range below or above the median of the group. If half or more data points collected from one animal within one brain region were outliers based on this criterion, the whole animal was excluded from analyses concerning that region. Another criterion for excluding an animal from any specific test was insufficient fixation (total number of animals included per brain region is indicated in Table 1). Distribution normality was assessed with the Shapiro-Wilk test. For regions where the data were normally distributed the groups were compared using independent *t*-tests, and for non-normally distributed regions using the Mann-Whitney *U*-test.

Correlations were assessed using Pearson’s *r*. To assess correlated activity between regions, for each brain region pair we included all animals with cell count data available for both regions. Correlation coefficients were calculated separately for control and defeated groups, pairing each brain region with each of the other brain regions.

All *p*-values were assessed against a two-tailed α-level of 0.05 to determine significance. SPSS Statistics version 24 (IBM) was used for all analyses. The Benjamini Hochberg procedure was used for correcting multiple comparisons for false discoveries^61^.

### Data availability Statement

All data are available from the corresponding authors upon request.

## Electronic supplementary material

Supplementary Figures

Supplementary Table S1
