# Supplementary material for: Brain activation induced by chronic psychosocial stress in mice
Source: Sci Rep. 2017 Nov 8;7:15061. doi: 10.1038/s41598-017-15422-5 (PMC5678090; doi:10.1038/s41598-017-15422-5)
Supplement: Supplementary file 1 — Supplementary Figures [file 41598_2017_15422_MOESM1_ESM.pdf]

Brain activation induced by chronic psychosocial stress in mice

Mikaela Antonina Laine[1], Ewa Sokolowska[1], Mateusz Dudek[2], Saija-Anita Callan[1], Petri Hyytiä[2]*, Iiris Hovatta[1]*

[1]*Department of Biosciences, University of Helsinki, Helsinki, Finland*

[2] *Department of Pharmacology, University of Helsinki, Helsinki, Finland*

SUPPLEMENTARY FIGURES

Supplementary Figure S1. Validation of primary FOSB antibody staining and correlation of ΔFOSB expression between brain regions. (**a**) Mice ($N = 2$/group) were administered either cocaine or saline by injections. Naïve non-injected mice were used as controls. Bars represent the mean number of cells staining positive for ΔFOSB in the caudate putamen in each group. Significant differences at $p < 0.001$ (independent *t*-test) are indicated by asterisks (***). Scale bars represent ±1 SEM. (**b**) Scatter plot of the mean ΔFOSB expression and mean value of *r* of each brain region, separately for control (light orange, $r = 0.633$, $p = 0.005$) and defeated (dark brown, $r = 0.162$, $p = 0.521$) groups.

Supplementary Figure S2. Representative images of immunohistochemical staining from each selected brain region. Micrographs captured at 20x magnification, displaying FOSB-positive cells stained with DAB (shown as maroon, see arrows on top row). Scale bar = 100 μm.

Supplementary Figure S1

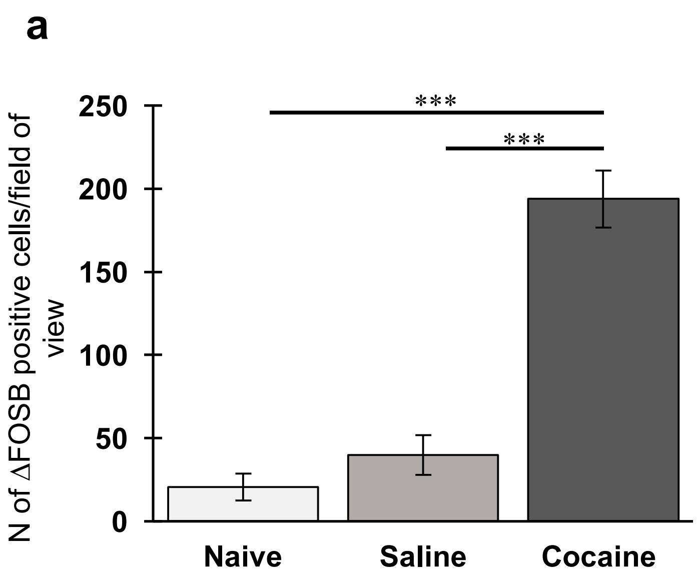

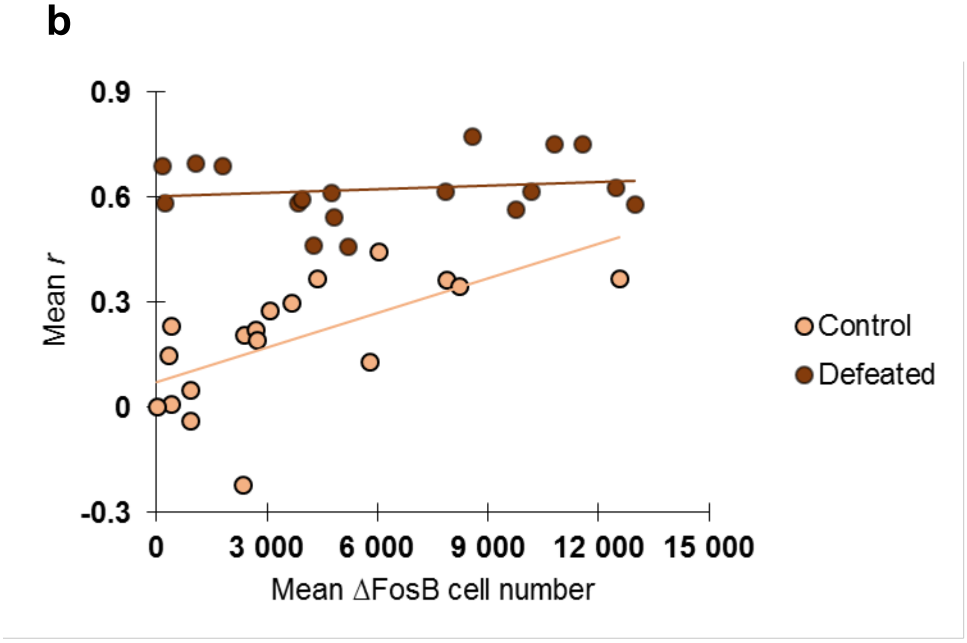

Supplementary Figure S2

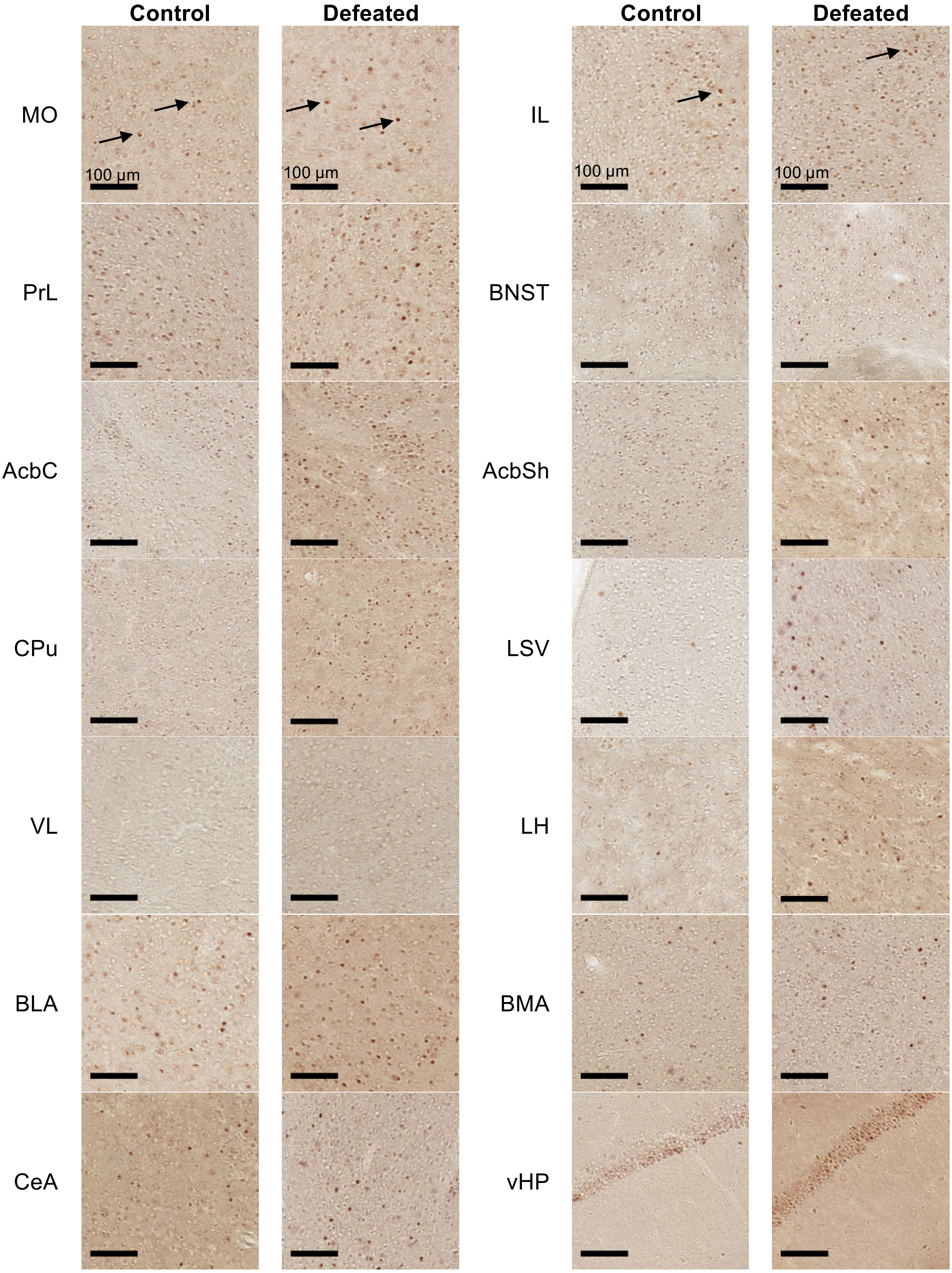

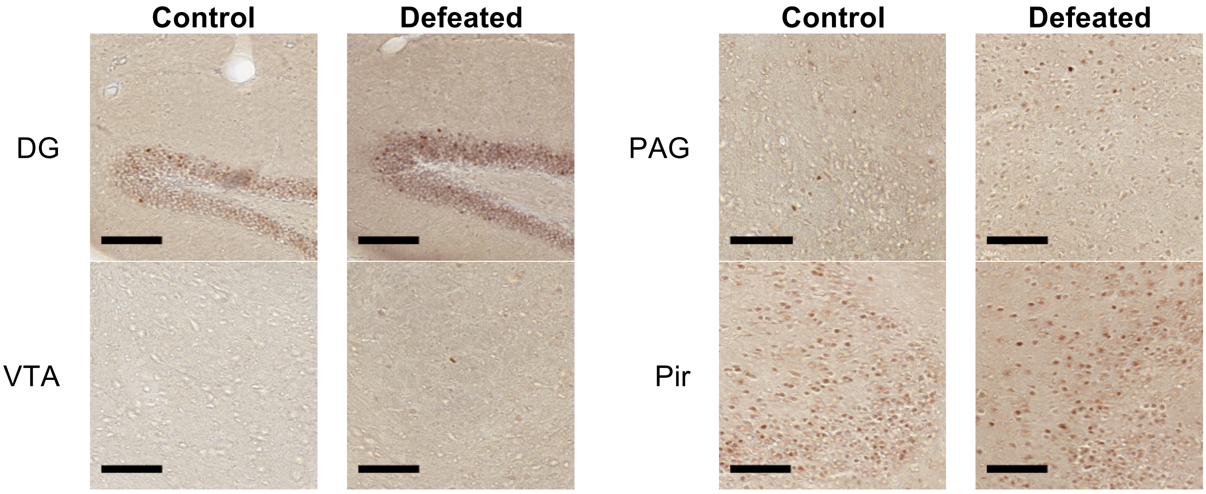
Control
Defeated
Control
Defeated
DG
VTA
PAG
Pir
