# Supplementary material for: Brain activation induced by chronic psychosocial stress in mice
Source: Sci Rep. 2017 Nov 8;7:15061. doi: 10.1038/s41598-017-15422-5 (PMC5678090; doi:10.1038/s41598-017-15422-5)
Supplement: Supplementary file 2 — Supplementary Table S1 [file 41598_2017_15422_MOESM2_ESM.pdf]

Brain activation induced by chronic psychosocial stress in mice

Mikaela Antonina Laine[1], Ewa Sokolowska[1], Mateusz Dudek[2], Saija-Anita Callan[1], Petri Hyytiä[2], Iiris Hovatta[1]

[1] Department of Biosciences, University of Helsinki, Helsinki, Finland

[2] Department of Pharmacology, University of Helsinki, Helsinki, Finland

Supplementary Table S1. Symmetrical correlation matrix for the number of ΔFosB-positive cells in each examined brain region, displayed on alternating rows for control and defeated animals. Values indicate Pearson's *r* , and in brackets and italizised the corresponding nominal *p* -value. Significant coefficients are indicated by asterisks.

Condition	Region	MO	IL	PrL	BNST	AcbC	AcbSh	CPu	LSV	VL	LH	BLA	BMA	CeA	vHP	DG	PAG	VTA	Pir
Control	MO	1,000																	
Defeated	MO	1,000																	
Control	IL	0.312 (0.496)	1,000																
Defeated	IL	0.821 (0.024)*	1,000																
Control	PrL	0.565 (0.186)	0.731 (0.062)	1,000															
Defeated	PrL	0.956 (0.001)**	0.939 (0.001)**	1,000															
Control	BNST	-0.357 (0.487)	-0.328 (0.526)	0.023 (0.966)	1,000														
Defeated	BNST	0.703 (0.119)	0.675 (0.096)	0.786 (0.036)*	1,000														
Control	AcbC	0.460 (0.299)	0.583 (0.169)	0.851 (0.015)*	0.265 (0.612)	1,000													
Defeated	AcbC	0.633 (0.127)	0.857 (0.007)**	0.718 (0.045)*	0.261 (0.572)	1,000													
Control	AcbSh	0.409 (0.421)	0.748 (0.087)	0.551 (0.257)	-0.690 (0.198)	0.654 (0.159)	1,000												
Defeated	AcbSh	0.589 (0.164)	0.581 (0.131)	0.590 (0.124)	0.225 (0.628)	0.740 (0.036)*	1,000												
Control	CPu	0.474 (0.283)	-0.358 (0.430)	0.090 (0.848)	0.470 (0.347)	0.230 (0.620)	-0.323 (0.532)	1,000											
Defeated	CPu	0.712 (0.073)	0.720 (0.044)*	0.734 (0.038)*	0.870 (0.011)*	0.409 (0.315)	0.089 (0.834)	1,000											
Control	LSV	0.038 (0.935)	0.194 (0.676)	-0.198 (0.671)	-0.333 (0.519)	-0.510 (0.242)	-0.291 (0.576)	-0.061 (0.896)	1,000										
Defeated	LSV	0.433 (0.332)	0.662 (0.073)	0.522 (0.185)	0.171 (0.714)	0.804 (0.016)*	0.373 (0.363)	0.341 (0.409)	1,000										
Control	VL	0 (n/a)	0 (n/a)	0 (n/a)	0 (n/a)	0 (n/a)	0 (n/a)	0 (n/a)	0 (n/a)	0 (n/a)									
Defeated	VL	0.893 (0.017)*	0.865 (0.012)*	0.853 (0.015)*	0.870 (0.024)*	0.711 (0.073)	0.657 (0.109)	0.877 (0.010)*	0.425 (0.342)	1,000									
Control	LH	0.458 (0.361)	0.196 (0.710)	-0.079 (0.881)	-0.865 (0.058)	-0.079 (0.882)	0.674 (0.212)	-0.283 (0.587)	-0.137 (0.795)	0 (n/a)	1,000								
Defeated	LH	0.762 (0.078)	0.575 (0.177)	0.772 (0.042)*	0.742 (0.092)	0.274 (0.552)	0.770 (0.043)*	0.488 (0.267)	0.154 (0.741)	0.631 (0.179)	1,000								
Control	BLA	0.735 (0.096)	0.310 (0.550)	0.673 (0.143)	0.075 (0.905)	0.843 (0.035)*	0.621 (0.264)	0.684 (0.134)	-0.451 (0.369)	0 (n/a)	0.213 (0.685)	1,000							
Defeated	BLA	0.789 (0.062)	0.486 (0.269)	0.718 (0.069)	0.647 (0.165)	0.225 (0.628)	0.697 (0.082)	0.467 (0.290)	0.187 (0.688)	0.539 (0.270)	0.970 (<0.001)**	1,000							
Control	BMA	0.749 (0.086)	-0.453 (0.366)	0.082 (0.877)	-0.168 (0.787)	0.263 (0.614)	0.124 (0.843)	0.493 (0.320)	-0.330 (0.523)	0 (n/a)	0.514 (0.297)	0.518 (0.292)	1,000						
Defeated	BMA	0.692 (0.128)	0.444 (0.319)	0.576 (0.176)	0.302 (0.561)	0.425 (0.342)	0.053 (0.910)	0.502 (0.251)	0.642 (0.120)	0.276 (0.597)	0.576 (0.176)	0.715 (0.071)	1,000						
Control	CeA	0.596 (0.212)	-0.419 (0.408)	0.261 (0.618)	0.440 (0.458)	0.265 (0.612)	-0.468 (0.426)	0.925 (0.008)*	0.127 (0.810)	0 (n/a)	-0.345 (0.503)	0.450 (0.370)	0.522 (0.288)	1,000					
Defeated	CeA	0.594 (0.214)	0.752 (0.051)	0.684 (0.090)	0.424 (0.402)	0.824 (0.023)*	0.025 (0.957)	0.644 (0.119)	0.856 (0.014)*	0.468 (0.350)	0.497 (0.256)	0.542 (0.209)	0.834 (0.020)*	1,000					
Control	vHP	0.293 (0.524)	0.077 (0.870)	0.575 (0.177)	0.412 (0.417)	0.545 (0.205)	0.139 (0.793)	0.598 (0.156)	-0.305 (0.505)	0 (n/a)	-0.361 (0.482)	0.729 (0.100)	-0.053 (0.920)	0.422 (0.405)	1,000				
Defeated	vHP	0.804 (0.029)*	0.789 (0.020)*	0.837 (0.010)*	0.575 (0.177)	0.500 (0.207)	0.164 (0.698)	0.662 (0.074)	0.560 (0.149)	0.522 (0.229)	0.606 (0.150)	0.596 (0.158)	0.715 (0.071)	0.853 (0.015)*	1,000				
Control	DG	0.353 (0.492)	0.844 (0.035)*	0.731 (0.099)	-0.037 (0.953)	0.641 (0.170)	0.666 (0.219)	0.301 (0.562)	-0.112 (0.832)	0 (n/a)	-0.007 (0.990)	0.700 (0.121)	-0.202 (0.701)	0.039 (0.941)	0.831 (0.040)*	1,000			
Defeated	DG	0.903 (0.036)*	0.821 (0.045)*	0.888 (0.018)*	0.965 (0.008)**	0.716 (0.110)	0.405 (0.425)	0.888 (0.018)*	0.781 (0.067)	0.926 (0.001)**	0.762 (0.078)	0.743 (0.091)	0.780 (0.067)	0.923 (0.009)**	0.750 (0.086)	1,000			
Control	PAG	-0.148 (0.752)	-0.504 (0.249)	0.125 (0.790)	0.701 (0.121)	0.200 (0.667)	-0.337 (0.514)	0.335 (0.463)	-0.694 (0.083)	0 (n/a)	-0.417 (0.410)	0.325 (0.530)	0.096 (0.856)	0.254 (0.627)	0.604 (0.151)	0.158 (0.765)	1,000		
Defeated	PAG	0.785 (0.036)*	0.954 (<0.001)**	0.876 (0.004)**	0.748 (0.053)	0.838 (0.009)**	0.592 (0.122)	0.796 (0.018)	0.627 (0.096)	0.954 (0.001)**	0.547 (0.204)	0.471 (0.286)	0.396 (0.379)	0.687 (0.088)	0.647 (0.083)	0.855 (0.030)*	1,000		
Control	VTA	0.633 (0.178)	-0.229 (0.662)	0.032 (0.952)	-0.471 (0.424)	0.219 (0.677)	0.489 (0.404)	0.135 (0.799)	-0.441 (0.382)	0 (n/a)	0.803 (0.054)	0.447 (0.374)	0.900 (0.014)*	0.109 (0.838)	-0.207 (0.694)	-0.160 (0.762)	-0.025 (0.962)	1,000	
Defeated	VTA	0.878 (0.050)	0.371 (0.469)	0.622 (0.187)	0.500 (0.391)	0.209 (0.692)	0.451 (0.370)	0.578 (0.229)	0.154 (0.771)	0.594 (0.291)	0.700 (0.121)	0.830 (0.041)*	0.903 (0.014)*	0.633 (0.177)	0.527 (0.282)	0.643 (0.168)	0.414 (0.415)	1,000	
Control	Pir	0.649 (0.115)	0.523 (0.228)	0.891 (0.007)**	0.191 (0.718)	0.843 (0.017)*	0.320 (0.537)	0.263 (0.569)	-0.260 (0.574)	0 (n/a)	-0.132 (0.803)	0.678 (0.139)	0.487 (0.328)	0.614 (0.195)	0.415 (0.355)	0.344 (0.505)	0.162 (0.728)	0.286 (0.583)	1,000
Defeated	Pir	0.839 (0.018)*	0.547 (0.161)	0.727 (0.041)*	0.470 (0.287)	0.503 (0.203)	0.832 (0.010)*	0.334 (0.419)	0.183 (0.664)	0.662 (0.106)	0.848 (0.016)*	0.870 (0.011)*	0.436 (0.329)	0.160 (0.733)	0.356 (0.387)	0.638 (0.173)	0.552 (0.156)	0.900 (0.015)*	1,000

MO: medial orbital cortex; IL: infralimbic cortex; PrL: prelimbic cortex; BNST: bed nucleus of stria terminalis (anterodorsal); AcbC: nucleus accumbens core; AcbSh: nucleus accumbens shell; CPu: caudoputamen; LSV: lateral septum (ventral); VL: thalamus (ventrolateral); LH: Hypothalamus (lateral); BLA: basolateral amygdala; BMA: basomedial amygdala; CeA: central nucleus of the amygdala; vHP: ventral hippocampus; DG: dentate gyrus (ventral); PAG: periaqueductal grey (dorsolateral); VTA: ventral tegmental area; Pir: piriform cortex
